# Proteomic evidences for microcystin-RR-induced toxicological alterations in mice liver

**DOI:** 10.1038/s41598-018-19299-w

**Published:** 2018-01-22

**Authors:** Ashutosh Kumar Rai, Rupesh Chaturvedi, Ashok Kumar

**Affiliations:** 1 School of Biotechnology, Institute of Science, Banaras Hindu University, Varanasi, 221005 India; 2 School of Biotechnology, Jawaharlal Nehru University, New Delhi, 110067 India; 3 Present Address: Department of Biochemistry, College of Medicine, Imam Abdulrahman Bin Faisal University, Dammam, 31441 Kingdom of Saudi Arabia

## Abstract

This study deals with the isolation and purification of an important variant of microcystins namely microcystin-RR (MCYST-RR) from *Microcystis aeruginosa* and reports its effects on mice liver protein profile and cellular functions. Protein profiling by 2-dimensional gel electrophoresis revealed changes in the number and accumulation of protein spots in liver of mice treated with different concentrations of MCYST-RR. Untreated (control) mice liver showed 368 protein spots while the number was 355, 348 and 332 in liver of mice treated with 200, 300 and 400 µg kg body wt^−1^ of MCYST-RR respectively. Altogether 102, 97, and 92 spots were differentially up-accumulated and 93, 91, and 87 spots were down- accumulated respectively with the treatment of 200, 300, 400 µg kg body wt^−1^. Eighteen differentially accumulated proteins present in all the four conditions were identified by MALDI-TOF MS. Of these eighteen proteins, 12 appeared to be involved in apoptosis/toxicological manifestations. Pathway analysis by Reactome and PANTHER database also mapped the identified proteins to programmed cell death/apoptosis clade. That MCYST-RR induces apoptosis in liver tissues was also confirmed by DNA fragmentation assay. Results of this study elucidate the proteomic basis for the hepatotoxicity of MCYST-RR which is otherwise poorly understood till date.

## Introduction

**Figure 1:**
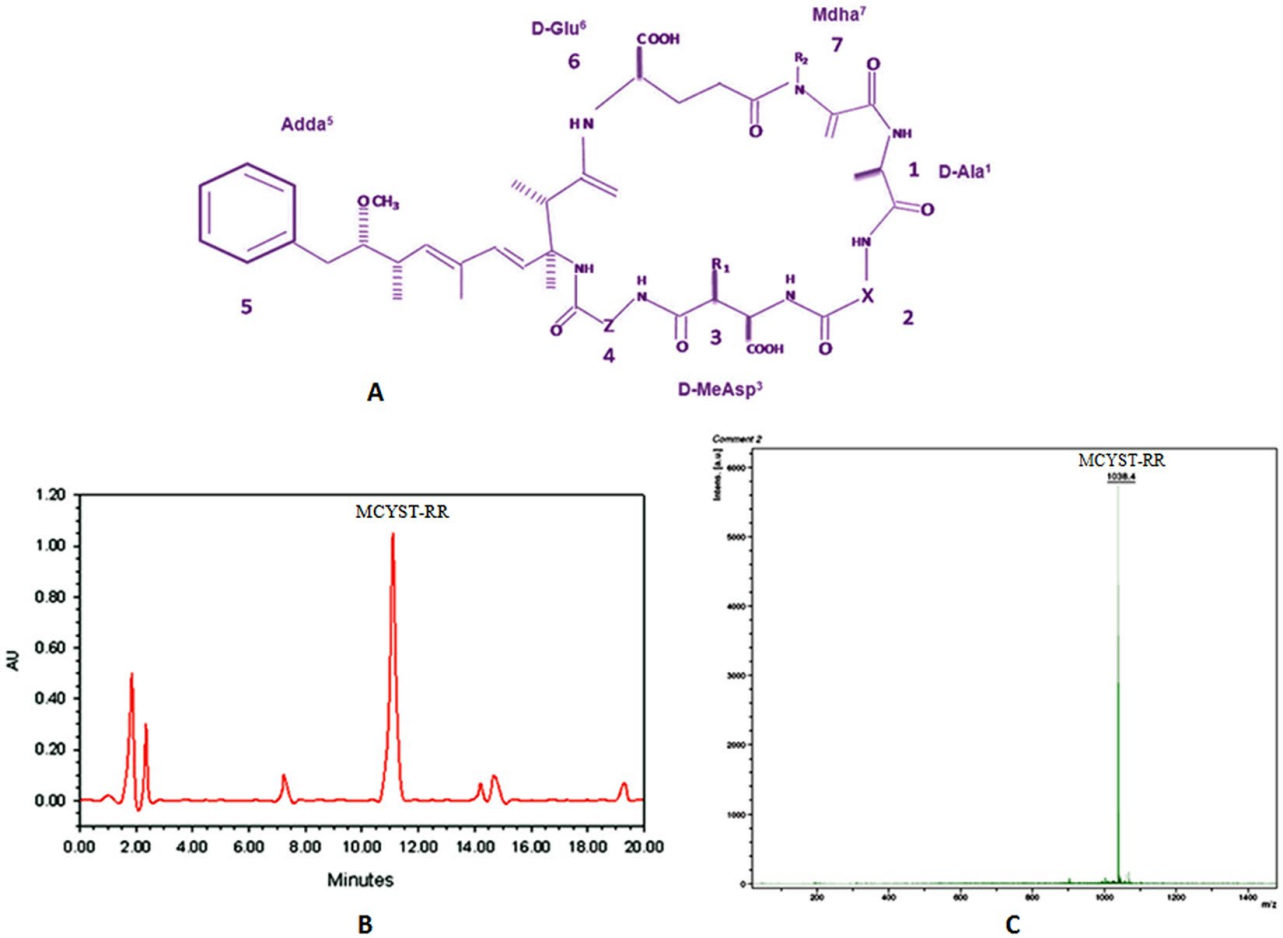
General structure of microcystins and purification of MCYST-RR. (**A**) General structure of heptapeptide microcystins. Variable L-amino acid residues are found at positions X and Z (modified from Rai *et al*.^14^), (**B**) HPLC chromatogram of toxins extracted from laboratory-grown unialgal *M*. *aeruginosa* strain V-08. Major peak with tr 11.1 min matches with MCYST-RR, and (**C**) MALDI-TOF MS chromatogram of HPLC purified MCYST-RR. Presence of a peak at m/z = 1038.4 (M + H^+^) confirms the presence of MCYST-RR. Toxins from *M*. *aeruginosa* were extracted as per the methods described in materials and method section.

Cyanobacteria (blue-green algae) inhabiting a wide spectrum of habitats including terrestrial, freshwater and marine are the most primitive oxygen-evolving Gram-negative photosynthetic prokaryotes^1^. Members of cyanobacteria show considerable metabolic plasticity and produce a variety of secondary metabolites including cyanotoxins and potential drugs^1–4^. Among cyanotoxins, the hepatotoxic microcystins (MCYSTs) are the most widely distributed low molecular weight cyclic heptapeptide synthesized by different genera of cyanobacteria including *Microcystis*, *Anabaena*, *Planktothrix*, and *Nostoc*^2,5–9^. Microcystins are represented by the general structure: cyclo- (D-Ala^1^-X^2^-D-MeAsp^3^-Z^4^-Adda^5^-D-Glu^6^-Mdha^7^) where X and Z are variable L-amino acids; MeAsp stands for erythro-β-methyl aspartate; Adda for a unique β-amino acid, (2 S, 3 S, 8 S, 9 S)-3-amino-9-methoxy-2, 6, 8-trimethyl-10- phenyldeca-4, 6-dienoic acid; and Mdha is *N*-methyl-dehydroalanine (Fig. 1A). Chemical variations in the structure of microcystins are very common and more than 100 variants of microcystins with molecular weight of 900-1100 Da have been reported in diverse species and water systems^9–13^. Notably, the majority of isolates belonging to species of above genera can synthesize one or more variants of MCYSTs^10,14,15^. At time heavy growth of *Microcystis* leads to the formation of blooms, which conglomerate as scum on the water surface^2,9,11^ and after the death and decay of cells large amount of cyanotoxins are released which not only affect the water quality but become toxic to eukaryotic organisms including humans^2,3,16,17^. Higher incidence of liver cancer in China was found associated with drinking water contaminated with cyanobacterial toxin including microcystin^18^. Similarly, the tragic death of 60 patients in Caruaru, Brazil, in 1996 was reported due to the contamination of water with microcystin which was used in a hemodialysis unit^19^.

In mammals, MCYSTs are selective for hepatic cells causing disintegration of the hepatocyte, liver necrosis, internal hemorrhage and apoptosis leading to death by hemorrhagic shock^16,20,21^. The toxic effect of microcystins is mainly linked to the irreversible inhibition of protein serine-threonine phosphatases^2,16^. At enzymatic level, MCYSTs are potent inhibitors of eukaryotic protein phosphatases PP1 and 2A^22,23^. In fact, inactivation of these phosphatases results in disruption of cytoskeleton due to phosphorylation, oxidative stress, mitogen-activated protein kinase (MAPK) deregulation, and DNA damages^24,25^. In addition to the role of PP1 and PP2 in toxicological manifestations, it has been reported that MCYST-LR exposure can cause apoptosis in mouse liver and DNA damage^24,26^.

The molecular mechanisms of liver toxicity in mice with the treatment of MCYSTs and its variants other than MCYST-LR are poorly explored. As most previous studies were limited to physiological/biochemical processes, the need to conduct detailed investigations employing transcriptomic and proteomic approaches to understand and reveal the exact mechanism (s) of toxicity in mouse exposed to MCYSTs cannot be over-emphasized. During the last two decades, proteome analysis employing 2-dimensional gel electrophoresis (2-DE) has become a powerful tool for visualizing many proteins synthesized in the cell and paved the way for understanding global changes in the protein profile^27–29^. A few workers have studied proteomic changes following MCYST treatment and reported alterations in the number and expression of proteins^24,27,30^. Proteomic approaches using 2-DE and peptide mass fingerprinting (PMF) revealed up and/or down-accumulation of several liver proteins of mouse and medaka fish after treatment of MCYST-LR^24,27,31,32^.

Till date studies on MCYST-mediated proteomic changes in the liver have been mostly conducted with the most common microcystin variant, MCYST-LR^24,32^, sporadic studies on other variants of MCYSTs which are otherwise equally important have been undertaken. Prompted by the above lacuna, we studied the changes in the proteome of the liver from the mice exposed to a toxic variant of microcystins, namely MCYST-RR employing 2-DE followed by MALDI-TOF MS analysis. We aimed to (a) isolate and purify MCYST-RR from *Microcystis aeruginosa*, (b) study the changes in the proteome of mouse liver following MCYST-RR treatment, and (c) assess the possible role of identified proteins in toxicity. Hopefully, the outcomes of this study may provide new insights for understanding how MCYST-RR can induce certain proteins and affect liver architecture leading to the death of mice.

## Results

### Purification of microcystin-RR (MCYST-RR) and toxicity test

Before assessing the impact of MCYST-RR on toxicity and protein profile of mice liver, toxins of laboratory grown *M*. *aeruginosa* strain V-08 were isolated and purified employing standard methods. Accordingly, crude toxin was subjected to HPLC analysis wherein seven peaks with retention time (RT) of 1.8, 2.2, 7.4, 11.1, 14.2, 14.7 and 19.3 min appeared in the chromatogram (Fig. 1B). All the seven fractions were collected separately for toxicity test. Two fractions showing RT at 11.1 and 14.2 min caused death of mice (Fig. 1B). Henceforth, identification of both the above fraction was made using standard microcystin variants (RR and -LR). HPLC analysis of both the fractions together with standard microcystin variants (RR and -LR) showed that the fraction having RT of 11.1 min resembled with the standard MCYST-RR and fraction with RT of 14.2 min to MCYST-LR. With a view to check the purity of HPLC purified MCYST-RR fraction, its spectroscopic analysis was performed which showed a single peak at 238 nm. The purity of HPLC purified MCYST-RR fraction was also tested employing the MALDI TOF MS analysis where a major peak at m/z value = 1038.4 (M + H^+^) was observed which confirmed the purity and identity of MCYST-RR (Fig. 1C). Unless otherwise stated all further experiments were performed with the HPLC purified MCYST-RR.

**Figure 2:**
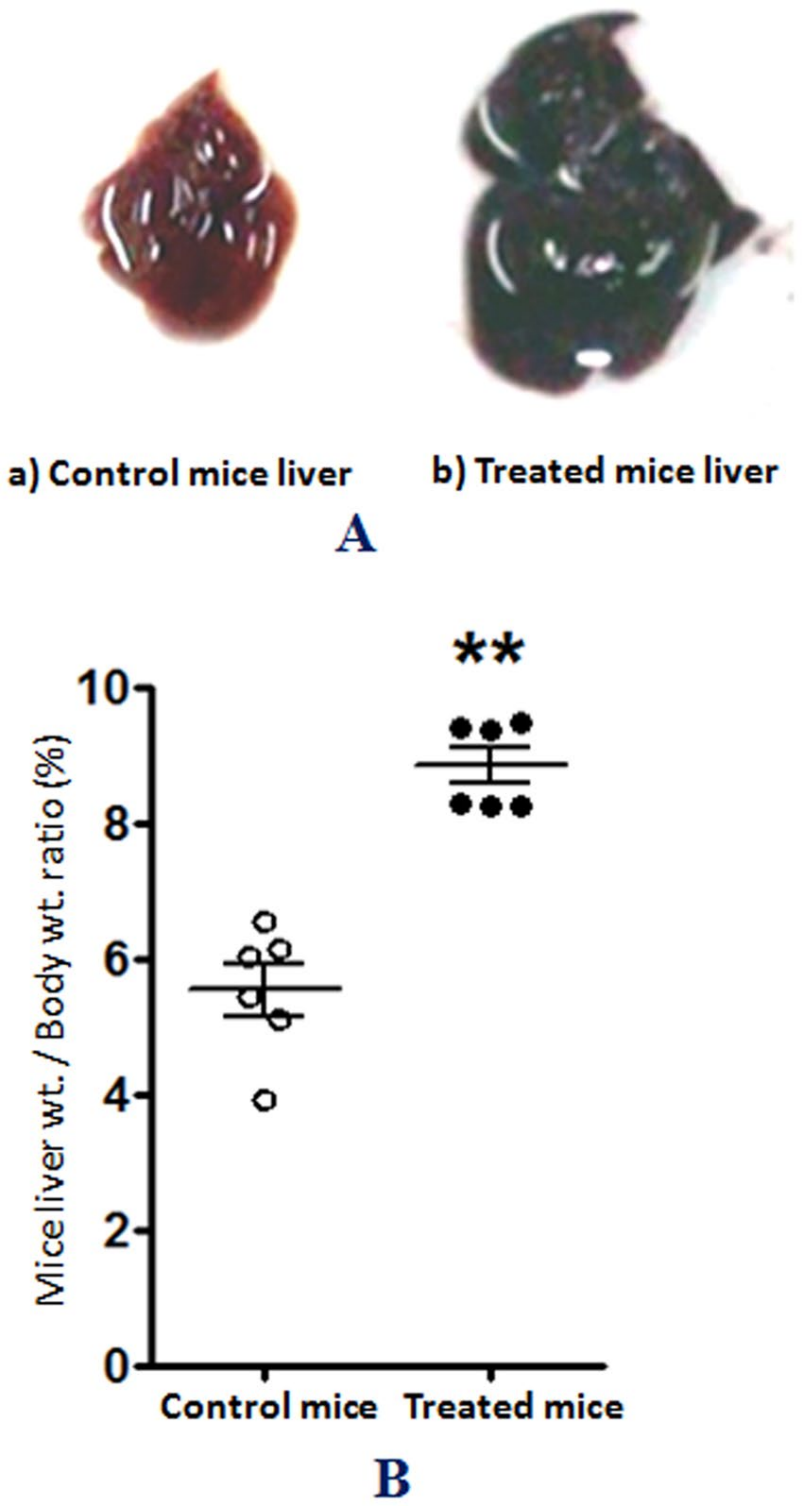
Effects of MCYST-RR on liver morphology and weight. (**A**)- a) Liver morphology and color of control mice (untreated), and b)- liver morphology of MCYST-RR treated mice. (**B**) Mice liver/body weight ratio under control (untreated) and MCYST-RR treated conditions. Mice were treated with 400 µg MCYST-RR kg body wt.^−1^ and sacrificed after 6 h. Liver of all the mice was aseptically excised and after visual examination for color and morphology, weight was taken and processed for histopathological investigation. Results are based on the average of three experiments performed independently under identical conditions.

After confirming the purity of MCYST-RR, mice bioassay was performed to test its toxicogenic property in a dose-dependent manner. MCYST-RR-treated mice (200, 300 and 400 µg kg body wt^−1^) showed clinical signs of poisoning such as restlessness, spasmodic leaping, fast breathing, slow movement, loss of co-ordination and splaying of hind limbs. Signs of poisoning and toxicity showed close correlation with increasing dose and duration of MCYST-RR treatment. Effects were more pronounced at 6 h with 400 µg kg body wt^−1^ of MCYST-RR administration. Symptoms of poisoning resembled to those caused by typical hepatotoxic microcystin*-*LR of *M*. *aeruginosa*. Detailed examination of the liver revealed significant increase in liver/body weight ratio and change in the color of the liver following the toxin administration (400 µg kg body wt^−1^) (Fig. 2A-b,B). The color of the liver changed to deep red due to hemorrhaging and blood pooling (Fig. 2A-b). Additionally, necropsy revealed a swollen liver and centrilobular to panlobular hemorrhagic necrosis. Excised liver of MCYST-RR-treated mice showed more than 1.5 fold increases in weight as compared to untreated control mice liver. Increased liver weight during manifestation of hepatotoxicity may be due to augmented inflammation leading to fluid retention in the organ and large scale hemorrhage resulting in accumulation of blood in the liver.

### MCYST-RR-induced alterations in liver protein profile

**Figure 3:**
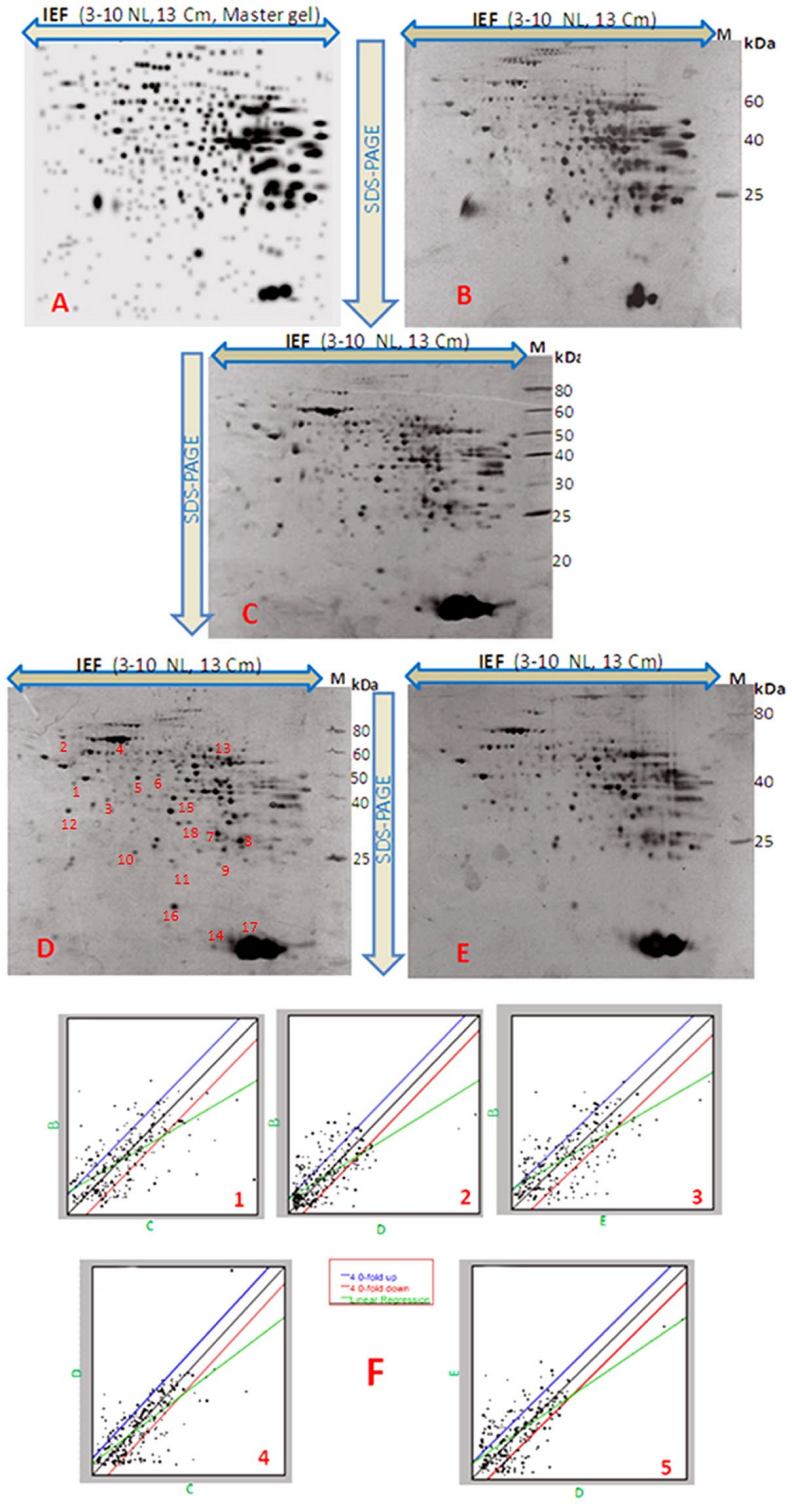
2-DE images of liver protein spots of mice following treatment of MCYST-RR for 6 h. (**A**) master gel generated by the PDQuest software, (**B**) 0 (untreated control), (**C**) 200, (**D**) 300, and (**E**) 400 µg MCYST-RR kg body wt.^−1^. (**F**) (1–5)-scatter plot showing level of expression of different spots of proteins. Spots showing up- and/or -down accumulation in 2-D gels are represented under different plots. Protein from liver was extracted as per the details described in the materials and method section. Equal amount of protein from all the sets of mice liver was loaded in each well. Other details as in Fig. 2.

**Figure 4:**
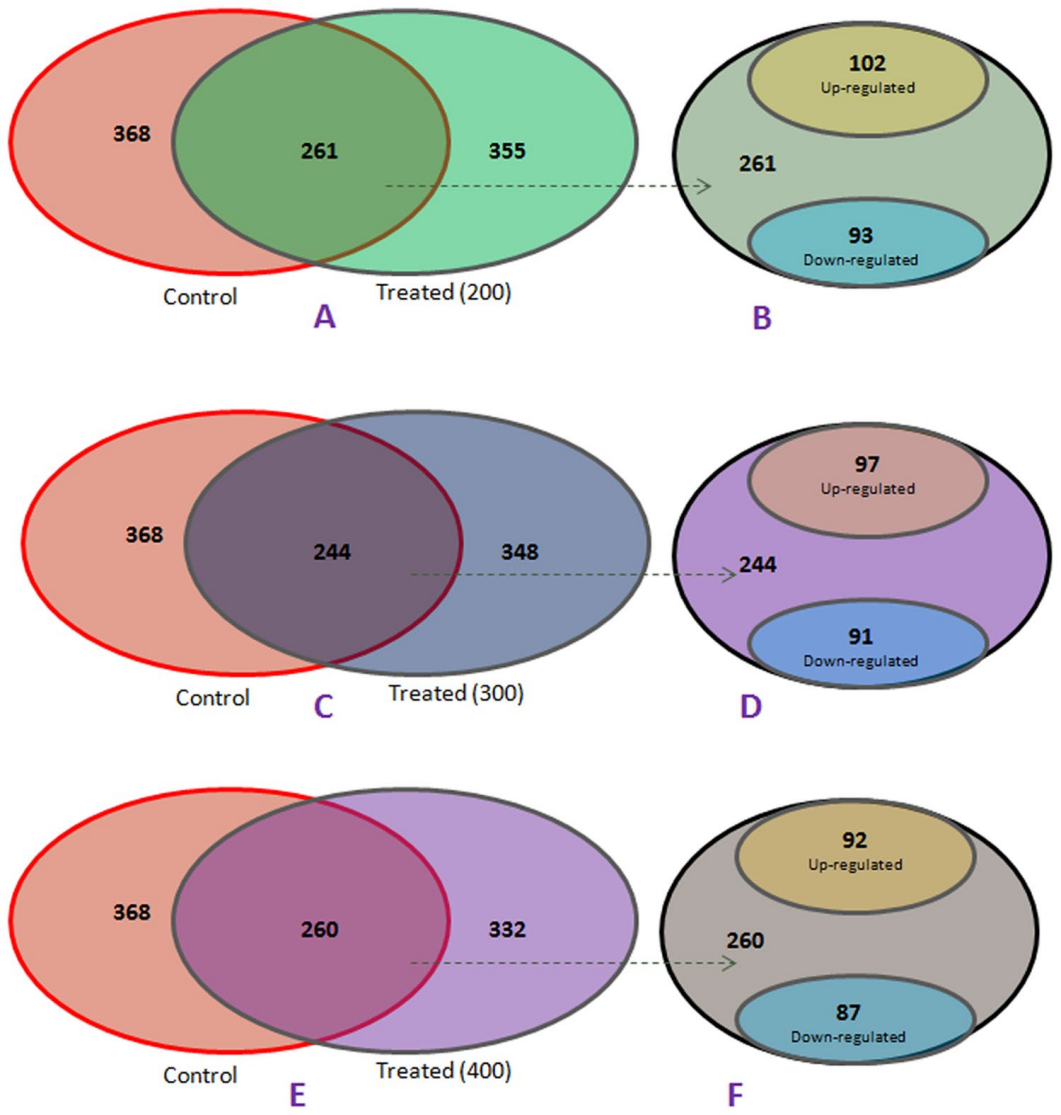
Venn diagram showing total and common protein spots in liver of toxin treated and control mice. Bubbles show common and up/down accumulated spots in liver of mice treated with varying concentrations of MCYST-RR. Number of common and up/down accumulated spots in control and treated mice is mentioned in the Figure.

Analysis of total proteome by 2-D gel electrophoresis of the liver of mice treated with different concentrations of MCYST-RR revealed significant alterations. It is evident from the analysis of gel (Fig. 3A–E) that changes in both number and intensity of protein spots occur upon administration of MCYST-RR to mice. Analysis of gels showed 368 protein spots in the liver of control mice (untreated) and 355, 348 and 332 spots in the liver of mice treated with 200, 300 and 400 µg kg body wt^−1^ of MCYST-RR for 6 h respectively (Fig. 3A–E). Further analysis of gels showed similarity in 261 protein spots (73%) of liver of mice treated with 200 µg kg body wt^−1^ of MCYST-RR with those of untreated control mice liver. Similarly, 244 spots (70%) of 300 µg and 260 spots (78%) of 400 µg kg body wt^−1^ treated sets were found similar to untreated (control) mice liver (Fig. 3A–E). Besides some changes in total number of spots, analysis of gels showed differential accumulation of several spots in mice liver proteins treated with different doses of MCYST-RR (Fig. 3F). Mice treated with 200 µg kg body wt^−1^ of MCYST-RR showed up-accumulation of 102 protein spots and down- accumulation of 93 spots as compared to untreated control. Similarly, treatment of 300 µg kg body wt^−1^ of MCYST-RR resulted in up-accumulation of 97 spots and down-accumulation of 91 spots. With 400 µg kg body wt^−1^ treatment of MCYST-RR, 92 proteins spots showed up-accumulation and 87 spots down-accumulation (Fig. 4A–F). Henceforth, our attention was focused mainly on the protein spots which were showing either up or -down accumulation as was evident from the spot-to-spot comparison.

### MALDI-TOF MS analysis and protein identification

**Figure 5:**
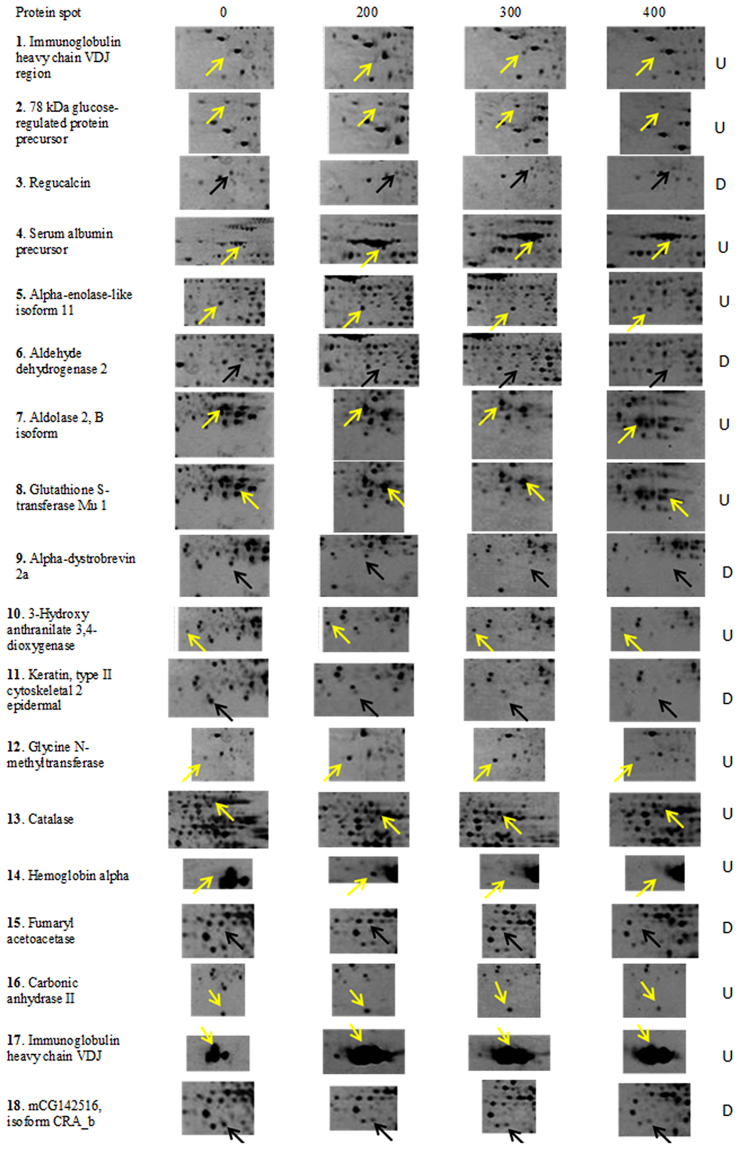
Accumulation pattern of selected proteins of liver following treatment of MCYST-RR to mice. Up- and/or -down accumulation of 18 selected protein spots upon treatment of 0, 200, 300, and 400 µg of MCYST-RR kg body wt^−1^ for 6 h to mice are denoted by arrows. Yellow arrows indicate up-accumulated spots (12) and black arrows indicate down- accumulated protein spots (6) in the conditions analyzed. Term ‘U’ denotes for up- accumulated proteins and ‘D’ denotes for down- accumulated proteins.

**Figure 6:**
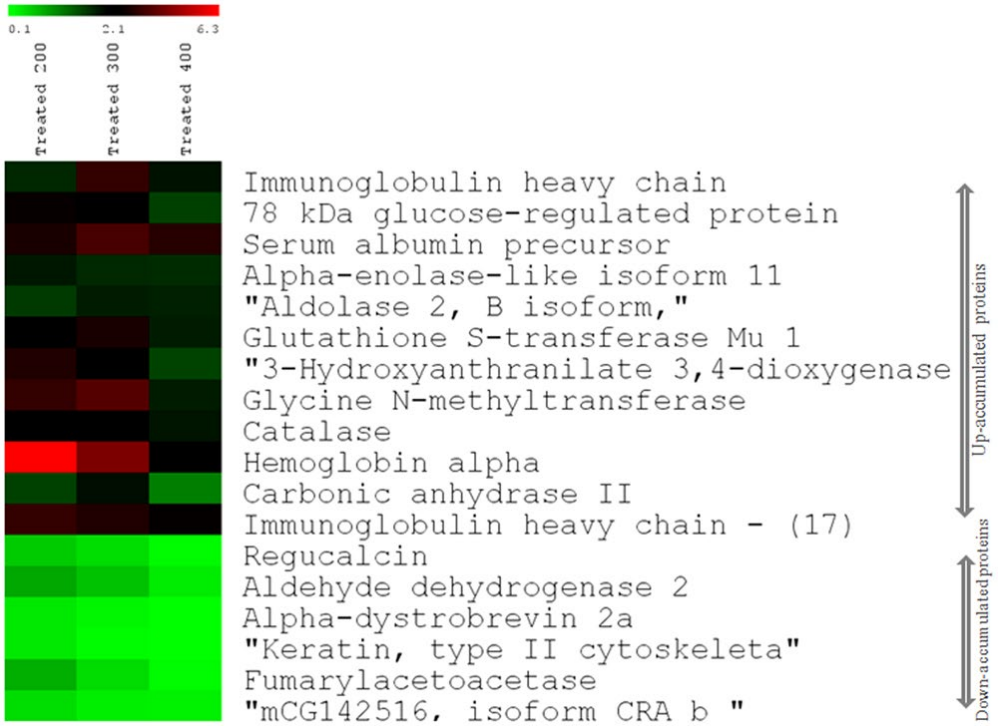
Heat map showing accumulation level of selected proteins in liver of mice following treatment of MCYST-RR (200, 300 and 400 µg kg body wt^−1^) for 6 h compared to liver of untreated control mice in a green (low relative expression) to red (high relative expression) scale. Name of 18 proteins is indicated on the right side of the Figure. Upper 12 proteins are up- accumulated and lower 6 are down-accumulated. Accumulation level of proteins is relative to untreated control mice proteins.

As the expression of several protein spots in liver was similar upon treatment with different concentrations of toxin to mice, we selected 18 spots which were common in all the four conditions of MCYST-RR treated sets of mice liver (0, 200, 300, & 400 µg MCYST-RR kg body wt^−1^). Of the 18 protein spots from 300 µg MCYST-RR kg body wt^−1^ treated set, 12 belonged to up-accumulated (1.71–6.3 folds) group and 6 to down-accumulated (0.62–0.21fold) group (Fig. 5). Proteins showing highest and significant level (p < 0.05) of matching with query proteins were analyzed using Mascot search engine through NCBInr database and identification of proteins was made. Amino acid sequences of a typical peptide from every protein spot have been provided along with their respective peaks (see Supplementary Fig. S1). All the spots were identified as different proteins as evident by their NCBI accession numbers and other specifications. Details such as expression level, sequence coverage, Mascot score, and numbers of peptides matched are presented in Table 1. To help visualize patterns in the expression level with various doses of MCYST-RR, we generated a heat map using all the 18 proteins (Fig. 6). Heat map indicated that 400 µg kg body wt^−1^ of MCYST-RR is overall cytotoxic. However, with 200 to 400 µg kg body wt^−1^ of MCYST-RR, a dose-dependent change in the levels of proteins was noticed as identified by proteomic approach.

**Table 1:** Details of protein spots identified by MALDI-TOF MS.

Spot No.	Accumulation (U/D)/Ratio to control*	Approx. PI/MW (kDa) on the gel	No. of peptides Matched	Mascot score of matching and significance level^#^	Sequence coverage (%)	Accession number	Predicted name of protein
1	U/3.1	5.2/50	4	67.9	56.1	gi|28875330	Immunoglobulin heavy chain VDJ region [*Mus musculus*]
2	U/2.0	4.5/75	9	71.6	24.8	gi|254540166	Glucose-regulated protein 78 kDa [*Mus musculus*]
3	D/0.43	5.5/32	11	83.2	35.1	gi|6677739	Regucalcin [*Mus musculus*]
4	U/3.4	6.0/70	7	70.4	18.8	gi|163310765	Serum albumin precursor [*Mus musculus*]
5	U/1.71	6.4/47	10	86.6	31.1	gi|309265190	Alpha-enolase-like isoform 2 [*Mus musculus*]
6	D/0.62	6.6/52	6	64.7	14.1	gi|148687772	Aldehyde dehydrogenase 2, mitochondrial, isoform CRA_a [*Mus musculus*]
7	U/1.8	9.0/30	14	113	59.6	gi|148670365	Aldolase 2, B isoform, isoform CRA_b [*Mus musculus*]
8	U/2.7	10/27	13	115	59.6	gi|6754084	Glutathione S-transferase Mu 1 [*Mus musculus*]
9	D/ 0.25	9.1/23	10	68.2	21.3	gi|4929247	Alpha-dystrobrevin 2a [*Mus musculus*]
10	U/2.2	6.3/26	8	65.6	23.2	gi|17921976	3-Hydroxyanthranilate 3,4-dioxygenase [*Mus musculus*]
11	D/0.21	7.8/23	7	66.3	12.9	gi|124487419	Keratin, type II cytoskeletal 2 epidermal [*Mus musculus*]
12	U/3.6	5.1/37	6	68.8	26.7	gi|55669634	Mouse glycine N-methyltransferase
13	U/2.03	9.0/58	16	135	29.4	gi|115704	RecName: Full = Catalase
14	U/4.2	9.0/15	7	103	78.9	gi|145301549	Hemoglobin α, adult chain 2 [*Mus musculus*]
15	D/0.44	7.8/42	11	86.1	31.7	gi|544273	RecName: Full = Fumarylacetoacetase
16	U/1.9	7.5/18	8	71.1	43.8	gi|309128	Carbonic anhydrase 2 [*Mus musculus*]
17	U/2.8	10/16	4	64.8	56.1	gi|28875330	Immunoglobulin heavy chain VDJ region [*Mus musculus*]
18	D/0.31	7.5/35	4	66	45.3	gi|148703101	mCG142516, isoform CRA_b [*Mus musculus*]

U- Up-accumulated, D-down- accumulated. *Based on quantification of data generated by PDQuest analysis between control Vs treated (300 µg kg body wt^−1^). Highest expression (6.3 times) with 200 µg kg body wt^−1^ treatment for the hemoglobin alpha. ^#^Significance level of matching was *p* < 0.05 for all the proteins.

### Pathways analysis for MCYST-RR induced toxicity

To investigate the pathways, we analyzed the data of 18 identified proteins in Reactome and PANTHER database. Reactome analysis mapped some proteins to the programmed cell death/apoptosis clade pointing to a specific node for apoptosis-induced DNA fragmentation (see supplementary Fig. S2-i). Additionally, a node of caspase activation via extrinsic signaling apoptotic pathway was also noted. From the detailed analysis of various nodes, it seems that induction of apoptotic process results in DNA fragmentation leading to cell death (see supplementary Fig. S2-i). Pathway deduced from Reactome analysis was found consistent with PATHER database which also showed an ‘apoptosis signaling pathways (Panther pathway accession P00006)’ having 72 components and 188 subfamilies (see supplementary Fig. S2-ii). Additionally, a survey of relevant literature also pointed that all these 12 proteins are directly or indirectly involved in the process of apoptosis (Table 2). However, the role of six proteins could not be linked to apoptosis by any source, their role in certain other cellular processes cannot be ruled out.

**Table 2:** Tentative role of identified proteins in apoptosis/toxicity.

Spot No.	Name of protein	Role in apoptosis (Yes/No)	Role of identified protein in apoptosis (from earlier reports)^•^	References
2	Glucose-regulated protein 78 kDa	Y	Novel mechanism of anti-apoptotic function of 78-kDa glucose-regulated protein reported recently in case of cancer	^53^
3	Regucalcin	Y	Inhibits PPase activity; over expression suppresses apoptotic cell death	^54^
4	Serum albumin precursor	Y	Specific inhibitor of apoptosis reported in endothelial cells	^55^
5	Alpha-enolase-like isoform 2	Y	A target of antibodies which induces cell death through an apoptotic process	^40^
6	Aldehyde dehydrogenase2, mitochondrial, isoform CRA_a	Y	Target of MCYST-LR other than PP1 & 2 A. Involved in acetaldehyde detoxification & prevention of free redical; over expression attenuate alcohol exposure induced apoptosis in liver.	^38^
8	Glutathione S-transferase Mu 1	Y	It modulates the stress activated signal by suppressing apoptosis signal regulating kinase 1 (As K1, which play critical role in cytokine and stress induced apoptosis	^41^
10	3-hydroxyanthranilate 3,4-dioxygenase	Y	Induces apoptosis in monocyte derived cell stimulated by interferon-gamma	^39^
12	Mouse glycine N-methyltransferase	Y	Major hepatic enzyme which regulate mediating methyl group availability in mammalian cells	^56^
13	RecName: Full = Catalase	Y	Catalase protect HepG2 cell from apoptosis induced by DNA damaging agents	^57^
14	Hemoglobin α, adult chain 2	Y	act as strong pro-apoptotic regulator	^37^
15	RecName: Full = Fumarylacetoacetase	Y	Tyrosenimia type 1 and apoptosis caused by deficiency of fumarylacetoacetate hydrolase (FAH) activity	^58^
16	Carbonic anhydrase 2	Y	Identified in autoimmune related pancreatitis	^59^

^•^Role of six identified proteins *viz*: Immunoglobulin heavy chain VDJ region (1), aldolase 2, B isoform, isoform CRA_b (7), alpha-dystrobrevin 2a (9), keratin, type II cytoskeletal 2 epidermal (11), immunoglobulin heavy chain VDJ region (17), and mCG142516, isoform CRA_b (18) in apoptosis is not available in database.

### MCYST-RR induces DNA fragmentation?

**Figure 7:**
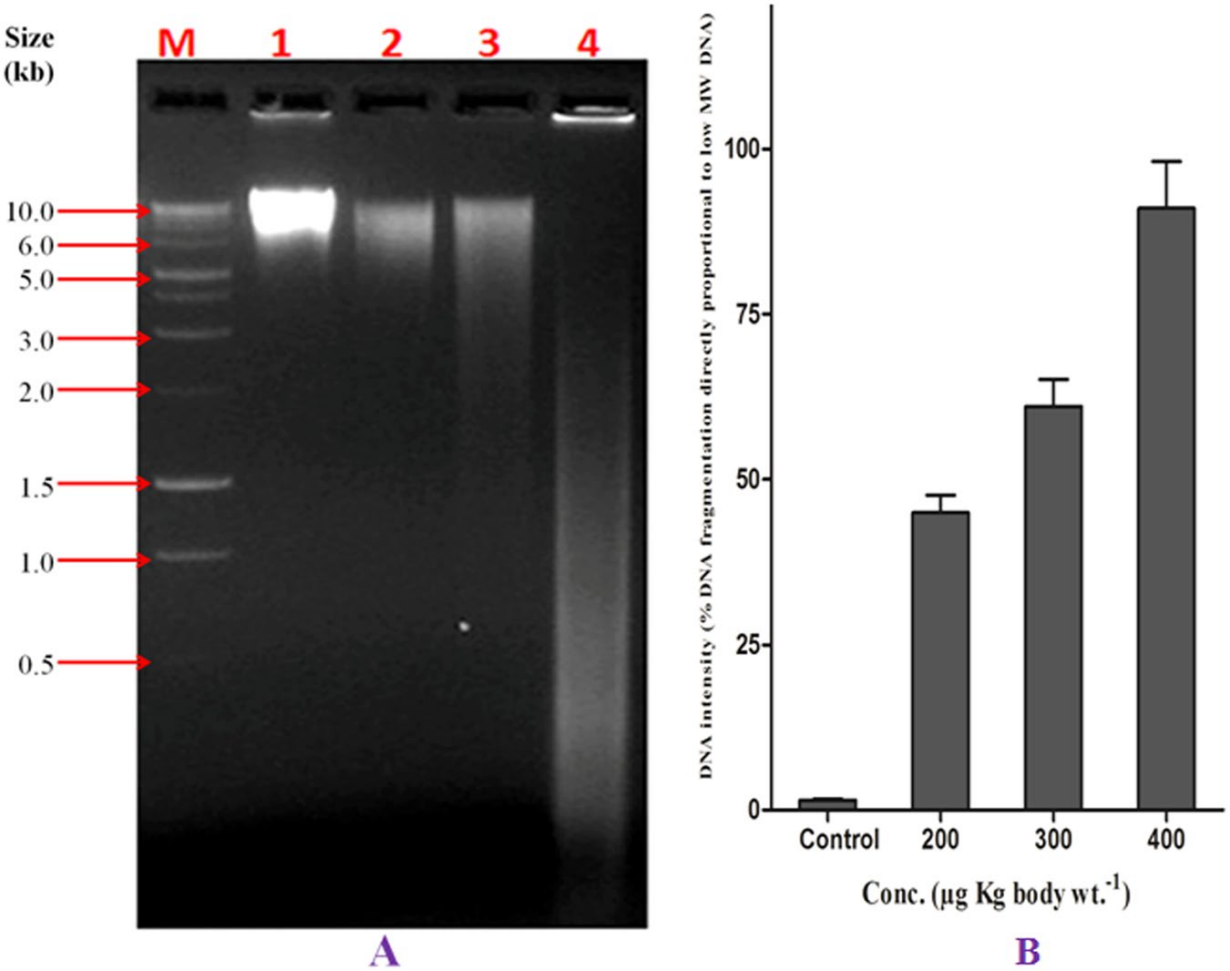
Effect of MCYST-RR on DNA fragmentation in mice liver. (**A)** agarose gel showing DNA fragmentation. M- Molecular weight marker (1 Kb DNA ladder), Lanes 1–4; 1- untreated control mice liver DNA, 2–200, 3–300, and 4–400 µg MCYST-RR kg body wt^−1^ treated mice liver DNA. Liver was excised from control and MCYST-RR treated mice (after 6 h) and DNA was isolated for fragmentation assay. (**B**) histogram showing changes in intensity along with % fragmentation of DNA of mice liver following treatment of varying concentrations of MCYST-RR. Value of % DNA fragmentation is proportional to the intensity of low molecular weight DNA. Equal amount of DNA from all the sets including control was loaded in each well. Test of DNA fragmentation was made in duplicate and repeated three times under identical conditions.

The administration of MCYST-RR to mice induced DNA fragmentation of the liver with all the concentrations tested. An internucleosomal fragmentation of DNA was observed with all the concentrations tested (200, 300 and 400 µg kg body wt^−1^ of MCYST-RR), the effect being more pronounced with 400 µg kg body wt^−1^ of toxin (Fig. 7A). It is evident from the data that there is a significant increase in the low molecular weight DNA vis-à-vis % DNA fragmentation with increasing concentrations of MCYST-RR treatment, the maximum increase in intensity and % fragmentation of DNA (ca 88%) attained with 400 µg kg body wt^−1^ of the toxin (Fig. 7B). On the contrary marked decline in intensity of high molecular weight DNA was noted with increasing concentrations of MCYST-RR. It should be noted that increase in intensity of low molecular wt. DNA is proportional to % DNA fragmentation. Above results clearly point to the induction of DNA fragmentation in the liver of mice upon MCYST-RR administration.

## Discussion

The occurrence of toxic cyanobacterial blooms from freshwater reservoirs and their acute toxicity to different organisms including animals and humans have been reported from different parts of the world^2,3,30,33–35^. Among the cyanotoxins produced by cyanobacteria, rapid advances have been made in the study of microcystins (MCYSTs) more specifically the most common variant MCYST-LR which shows acute toxicity^11,17,26^. The main aim of this study was to assess the effects of an equally important variant of MCYSTs namely MCYST-RR on liver protein profile. The study resulted in the isolation and purification of MCYST-RR from a fresh water bloom-forming *M*. *aeruginosa* strain V-08 which has not been reported till date from this part of India. The identity of MCYST-RR was based on the data of HPLC analysis where a peak identical to standard MCYST-RR was noticed with a retention time of 11.1 min. Furthermore, MALDI-TOF MS analysis of the purified MCYST-RR also showed a single major peak at m/z value = 1038.4 (M + H^+^). Above findings are similar to earlier reports where mass spectra of MCYST-RR from a few cyanobacterial samples including *Microcystis* bloom showed m/z value in the range of 1024.6–1045.6^10,36^. That MCYST-RR isolated from *M*. *aeruginosa* strain V-08 is indeed an variant of MCYST was also evident from the clinical signs of poisoning in mice that resembled to those caused by the most common variant MCYST-LR^2^. As such our finding related to the isolation and purification of MCYST-RR is not new, nevertheless points to the widespread occurrence of MCYST-RR together with MCYST-LR in the bloom-forming toxic cyanobacterium *M*. *aeruginosa* from water bodies of India.

Several researchers have reported that MCYST-LR-induced liver lesions including necrosis, internal hemorrhage, cellular hypertrophy, and glycogen depletion are caused mainly due to the inhibition of protein phosphatase PP1 and PP2 activity^16,21,23^. Additionally, inhibition of PP1 and PP2 activity also induces apoptosis and dissociation of liver sheets^24^. However, the exact apoptotic mechanism (s) induced by MCYSTs and the roles of proteins/molecules that may be involved in apoptotic events have remained poorly understood till date. In this study, proteomics approach was employed to study alterations in the proteome of the liver of mice exposed to MCYST-RR with a view to understanding their role, if any, in apoptosis. Findings based on 2-DE revealed significant changes in the proteome of toxin-treated mice liver as compared to untreated (control) set. While alterations in the number of proteins were not very significant but marked differences in the level of accumulation of various proteins were noticed. As such changes in protein profile including number and quantity are expected as it is an essential step of adaptive/survival mechanism developed by any organism exposed to toxicants^32^. Similar to our observation, alterations in protein profile by the most prominent toxin variant MCYST-LR have been reported in medaka fish and mouse liver^27,31,32^. In an interesting report, apoptotic effect of oral administration of MCYST-RR has been reported in mice liver^21^. Above findings allow us to suggest that the effects of MCYST-RR on liver proteome may be similar to those exhibited by MCYST-LR. Such a conclusion is further supported by the identification and functions of 18 differentially accumulated protein spots by MALDI-TOF MS of the mice liver subjected to MCYST-RR treatment. Of the 18 differentially accumulated proteins, changes in the expression of glucose-regulated protein 78 kDa, aldehyde dehydrogenase 2 mitochondrial, hemoglobin α, glutathione S-transferase, carbonic anhydrase 2, regucalcin, fumarylacetoacetase, immunoglobulins and certain others have also been reported in mice with MCYST-LR treatment. However, 10 proteins namely, serum albumin precursor, alpha-enolase-like isoform 11, aldolase 2, B isoform, isoform CRA_b, alpha-dystrobrevin 2a, 3-hydroxyanthranilate 3,4-dioxygenase, keratin, type II cytoskeletal 2 epidermal, mouse glycine N-methyltransferase, catalase, immunoglobulin heavy chain VDJ region, and mCG142516, isoform CRA_b were specifically differentially accumulated upon MCYST-RR treatment. This suggests that in spite of significant similarity in the mode of action of MCYST-LR and MCYST-RR, some differences in the protein accumulation of liver do occur upon its administration to mice. Further work is needed to assign the exact role of these differentially accumulated proteins in hepatotoxicity of mice.

That the MCYST-RR may be involved in the induction of apoptosis is evident from the assigned functions and available literature showing the role of identified proteins in the process of apoptosis^27,31,32,37,38^. Modifications in terms of accumulation (up or -down) of these proteins may be one of the early and crucial events in the regulation of apoptotic event. Among the six down-accumulated proteins identified in this study, three proteins namely regucalcin, aldehyde dehydrogenase, and fumarylacetoacetase seem to be involved in apoptosis. MCYST-LR-induced down-accumulation of aldehyde dehydrogenase has been reported to cause MAPK deregulation, oxidative stress, DNA damages, and disruption of the translation and maturation of proteins^32^. Furthermore, it has been reported that aldehyde dehydrogenase-2 attenuates chronic alcohol exposure-induced apoptosis in liver^38^. Equally important protein regucalcin is known to play a multifunctional role in the regulation of cellular function in important organs including liver and kidney cortex. It has been reported that regucalcin can inhibit protein kinase, protein phosphatase, and nucleic acid and its down regulation may lead to cell death and apoptosis^24^. Our finding is in agreement with earlier report wherein down regulation of regucalcin was noted in mouse liver by MCYST-LR treatment and its role was implicated in the induction of apoptosis^24^.

Among the 12 proteins belonging to up-accumulated group, nine proteins seem to be involved in the process of apoptosis. Such a statement is supported by the findings of other researchers wherein the role of these differentially accumulated proteins in the induction of apoptosis upon MCYST-LR exposure to mice has been reported. To this effect, the expression of alpha globin was shown to be up-accumulated upon specific apoptotic stimuli like cytokine deprivation or cisplatin treatment in the hematopoietic pro-B cell line, FL5.12. It was also reported that the pro-apoptotic effect of hemoglobin was suppressed by Bcl-2 and there was enhanced stimulation of caspase activity^37^. Similarly, up-accumulation of 3-hydroxyanthranilate 3,4-dioxygenase in this study may have role in apoptosis as it promotes apoptosis in monocytes/macrophages under inflammatory or other pathophysiological conditions^39^. The role of alpha-enolase-like isoform 2 and glutathione S-transferase has also been implicated in apoptosis by a few researchers^24,40,41^. A few antiapoptotc proteins such as serum albumin precursor, catalase, and glucose-regulated protein 78 kDa were also up- accumulated whose role may be in imparting defense against toxin-induced toxicity in mice. A detailed study is needed to reveal the exact role of above proteins in apoptosis so as to derive a firm conclusion. However, the proteomic modifications induced by the treatment of the MCYST-RR in the present study are more or less similar to those induced by MCYST-LR^24,27,31,32^. Furthermore, the toxicity mechanism of MCYST-RR seems similar to that of MCYST-LR and induction of apoptosis seems very important in the toxicological manifestations. Our findings are consistent with MCYST-LR-induced apoptosis in mice and other higher organisms as reported by a few researchers^21,24,42–45^.

That the process of apoptosis is indeed involved in toxicity was also evident from the results of DNA fragmentation in the liver. Results showed intense DNA fragmentation in the liver cells following MCYST-RR treatment. Till date, no report exists on the effect of MCYST-RR on DNA fragmentation although DNA fragmentation by MCYST-LR has been reported^24^. This implies that the effect of MCYST-RR on DNA fragmentation is identical to MCYST-LR. That the event of apoptosis does take place was further corroborated by the Wright-Giemsa staining where the apoptotic cells were observed in bulk in liver of mice after MCYST-RR treatment. MCYST-RR treatment (400 µg kg body wt^−1^ for 6 h) increased the number of cells undergoing apoptosis, there were drastic changes in the architecture of liver cells including all the signs of apoptotic cells.

Altogether findings of this study show that MCYST-RR brings about significant changes in protein profile of liver and a few proteins are involved in the induction of apoptosis. Although we could not identify all the differentially accumulated proteins of the liver following MCYST-RR treatment but sufficient evidences showing alterations in protein profile and the role of certain proteins in the induction of apoptosis have been provided. It would be worthwhile to make a detailed analysis of all the MCYST-RR responsive proteins as well as assay of enzymes of the apoptotic pathway so as to better understand the mechanism of toxicity of this important variant of MCYST.

In summary, we demonstrate that administration of microcystin-RR isolated from the bloom-forming cyanobacterium *M*. *aeruginosa* V-08 to mice causes toxicity and alterations in the proteome of liver. Analysis of proteome of liver from toxin-treated mice showed alterations both in the number and expression of proteins. Eighteen differentially accumulated protein spots were identified by MALDI-TOF MS of which twelve were found involved in apoptosis. That these proteins indeed induce apoptosis was also evident from the DNA fragmentation assay and/Wright-Giemsa staining of liver cells and the pathway analysis by Reactome and PANTHER database which mapped the identified proteins to programmed cell death/ apoptosis. It would be worthwhile to make detailed analysis of all the differentially accumulated proteins so as to better understand their role (s) in toxicological manifestations. However, identification of differentially up and/or down accumulated proteins in this study may prove useful in future studies especially for assessing their significance in the toxicity mechanism of mice exposed to MCYST-RR and/or other variants of microcystins.

## Methods

### Test organism, growth conditions and maintenance

*M*. *aeruginosa* V-08 was isolated from a eutrophic pond of Varanasi city namely Pishach Mochan (25° 20′N and 83° 00′E). Bloom of *M*. *aeruginosa* was collected from Pishach Mochan pond and strains of *M*. *aeruginosa* present in the sample were isolated and purified employing standard microbiological techniques. One isolate showing better growth was characterized in detail and identified by 16S rRNA gene sequencing and accession number was obtained (JF799854)^30^. This isolate was designated as *M*. *aeruginosa* V-08. It was routinely grown in modified Parker’s medium^46^ in a culture room at 27 ± 2 °C and illuminated with cool white Sylvania 40 W T12 fluorescent tubes at an intensity of 14.4 ± 1 Wm^−2^ for a 14/10 h light/dark cycle. Unless otherwise stated all the experiments were performed by using *M*. *aeruginosa* V-08.

### Extraction of microcystin-RR (MCYST-RR)

MCYST-RR was extracted according to the modified method of Lawton and Edwards^47^. Briefly, 25 g lyophilized cells of *M*. *aeruginosa* V-08 were suspended in 250 mL of 70% aqueous methanol and sonicated in a Branson sonifier 450 (Branson Ultrasonics Corp., USA) for 3 min at 8 output and 60% duty cycle by keeping samples in an ice bath. After 2–3 cycles of sonication, the cell extract was stirred for 5 h at room temperature. Thereafter, the suspension was centrifuged at 12,000 × g for 20 min and the supernatant was collected. Pellet was re-extracted twice for complete recovery of toxins and the supernatant was pooled together, vacuum dried and dissolved in 5 mL of 10 mM ammonium acetate. Insoluble material, present, if any, was removed by centrifugation at 12,000 × g for 20 min and the supernatant obtained was evaporated to dryness under a stream of N_2_. The dried material (~300 mg) was dissolved in 5 mL HPLC grade methanol and centrifuged at 12,000 × g for 20 min for HPLC analysis.

### Purification of MCYST-RR by high performance liquid chromatography (HPLC)

MCYST-RR was separated and purified by HPLC (Waters Alliance 2695 solvent delivery system) equipped with a photodiode array (PDA) detector (2996 PDA, Waters). Separation was accomplished on a Sunfire C-18 column (2.1 mm × 150 mm) in gradient mode using water (Milli-Q) and acetonitrile, both containing 0.05% triflouroacetic acid (TFA) as a mobile phase. Samples were run at a flow rate of 1.0 mL min^−1^ and the separation was monitored with a variable UV detector operated at 238 nm. Instrumental control, data acquisition, and processing were achieved using Empower 2.0 software. The purity and identity of MCYST-RR were checked and confirmed by retention time and comparison of spectra with standard MCYST-RR (Sigma-Aldrich, MO, USA). Standard MCYST-LR (obtained as a gift from Prof. W.W. Carmichael, Wright State University, OH, USA) was also used for cross-examination. MCYST-RR purified by HPLC was stored at 4 °C in the dark for further studies. Quantification of MCYST-RR was done by measuring its specific absorbance at 238 nm using standard MCYST-RR and protein phosphatase 1 (PP1) inhibition assay^9^. Additionally, ELISA-based ‘QuantiPlate Kit for Microcystins’ (Envirologix Inc., Portland, USA) was used to confirm the data. This kit of MCYSTs is a competitive ELISA and widely used for the detection and quantification of microcystins in water samples.

### Determination of peptide mass

MALDI-TOF MS analysis was performed for the determination of the mass of peptide of purified MCYST-RR without tryptic digestion. Analyses were performed in the positive-ion mode, giving mainly singly protonated molecular ions [M + H^+^]. MALDI spectra were acquired for high resolution in a wide range of m/z value 0–2200 Da. Other details are similar to those described below under the section protein identification.

### Toxin treatment and toxicity test

Six to eight-weeks old disease-free male mice (Albino BALB/c Park’s strain) of 20 ± 2 g were selected for the toxicological studies^48^. Mice were divided into four groups (3 mice in each group) and purified MCYST-RR (0, 200, 300, and 400 µg kg body wt^−1^) was injected intraperitoneally (i.p.) in mice of each group over a period of 6 h. Selection of above dose of MCYST-RR treatment to mice was based on the fact that lower doses (50 and 100 µg kg body wt^−1^) did not show pronounced clinical signs of poisoning even after 4 h of toxin administration. Mice were sacrificed by cervical dislocation, and liver of all the mice was aseptically excised. The choice of i.p. injection of MCYSTs was based on the fact that they localize in the liver due to rapid uptake by hepatocytes and show instant signs of toxicity. On the other hand, due to poor absorption, orally administered MCYSTs are less toxic, signs of toxicity are delayed and lethal dose in mice is much higher as compared to i.v. or i.p route of administration^49^. Mice were kept and maintained in the animal room of the School of Biotechnology, Banaras Hindu University. This study was approved by the institutional ethics committee of Banaras Hindu University (Ref. No. Dean/2008-09/319 dated Jan. 05, 2009). All the experiments were performed as per the approved guidelines of ethical committee.

### Protein extraction

Whole livers excised from mice treated with MCYST-RR and control (untreated) were rinsed thrice with ice-cold phosphate-buffered saline (PBS), homogenized and suspended in sterilized MQ water (5 mL) containing 30 mM DTT. Freezing and thawing of suspension were done thrice to release the proteins. The resulting suspension was centrifuged at 15,000 × *g* at 4 °C for 20 min. The supernatant was transferred to a fresh tube and chilled 10% TCA in acetone was added and kept at -20 °C for 3 h for precipitation of proteins. Precipitated protein was collected by centrifugation at 15,000 × *g* at 4 °C for 30 min. The pellet obtained was rinsed twice with chilled acetone (95%) and after drying dissolved in rehydration buffer containing 7 M urea, 2 M thiourea, 2.5% (w/v) CHAPS, 0.3% (w/v) DTT, and 0.55% IPG buffer (GE Healthcare Bio-Sciences AB, Sweden). After proper dissolution of the sample in rehydration buffer, it was centrifuged at 12,000 × *g* at 4 °C for 20 min and the supernatant was collected. The protein content in each sample was measured by the Bradford method^50^.

### 2-Dimensional gel electrophoresis (2-DE) and spot detection

The first dimension isoelectric focusing (IEF) was performed using Immobiline DryStrip (pH 3–10, 13 cm) (GE Healthcare Bio-Sciences AB, Sweden). Protein sample (250 μg) dissolved in rehydration buffer was loaded onto each IGP strip and rehydrated for 14 h at 4 °C in the dark. IEF was performed using the Ettan IPGphor 3 IEF system (GE Healthcare, UK) at 20 °C using the following steps; 100 V for 2 h, 200 V for 2 h, 500 V for 2 h, 500 V for 2 h, 1500 V for 2 h, 3000 V for 2 h, 6000 V for 2 h, and 8000 V until a total of 55000 Vh was attained. Focused strips were immediately equilibrated twice in SDS equilibration buffer [50 mM Tris/HCl pH 8.8; 6 M urea; 30% (v/v) glycerol; 2% (w/v) SDS] containing; a) 1% (w/v) DTT in first part, and b) 2.5% (w/v) iodoacetamide in second part. Strips were placed for 20 min initially in first part of SDS equilibration buffer containing DTT followed by 20 min in second part containing iodoacetamide. After equilibration, the second dimension separation was carried out on 12% SDS-PAGE gel at 20 mA gel^−1^ using SE 600 Ruby multiple gel- electrophoresis system (GE Healthcare, UK). Experiments were done in triplicate for each protein sample of MCYST-RR treated and untreated control samples. Gels were stained with Colloidal Coomassie Blue G-250^51^ and images were captured using the AlphaImager Gel Documentation unit (Alpha Innotech, USA).

The gels were scanned and protein spots analyzed by PDQuest software version 8.0.1 (Bio-Rad Laboratories, USA) for spot detection, quantification, background subtraction and spot matching between various gels. Spot quantification in the control and treated gels (from toxin-treated mice) was performed by spot volumes (intensity × mm^2^) as described by Agrawal *et al*.^28^. The relative spot volume with respect to the toxin-treated and untreated control samples was compared by means of Student’s t-test. P values less than 0.05 were considered statistically significant.

### Trypsin digestion of protein spots

Desired spots were excised from the gel by manual picking using sterile OneTouch spot picker and placed into separate microcentrifuge tubes, and sliced into small pieces and washed twice with 50% acetonitrile made in 25 mM ammonium bicarbonate pH 8.0 to destain and thereafter soaked in 100% acetonitrile for 5 min to dehydrate. Sliced gel pieces were dried in a vacuum centrifuge for 20–30 min. Gel slices containing proteins were digested with 15 μL cold trypsin solution (Sigma Sequencing Grade) and incubated at 37 °C overnight followed by soaking in 25–50 μL of 50% acetonitrile in 5% TFA for 30–60 min with gentle agitation. The supernatant obtained was lyophilized and reconstituted by adding 3.0 μL of 50% acetonitrile in 0.1% TFA to the bottom of the tube followed by gentle pipetting to dissolve the extracted peptides.

### Identification of proteins

Protein spots were analyzed in an Ultraflex TOF/TOF Mass Spectrometer (Bruker Daltonics Inc., USA) at the Interdisciplinary School of Life Sciences, Banaras Hindu University, Varanasi. Mass spectra were recorded in the positive ion mode. MALDI spectrum was acquired with an optimized method for high resolution in the range of m/z value of 0–4500 Da. The proteins were identified by comparing peptide mass fingerprints at the NCBInr database using the Mascot search engine (http://www.matrixscience.com). Parameters set for the identification of proteins were: type of search-peptide mass fingerprint; data base- NCBInr and species *Mus musculus;* peptide masses [M + H^+^] and monoisotopic; fixed modifications-carbamidomethyl (C); variable modifications-oxidation (M); mass tolerance ±100 ppm; enzyme- trypsin. Identification of the matched proteins showing highest Mascot protein scores and best significance level of matching (*p* < 0.05) was established on the basis of the observed molecular weight and pI on the 2-DE gel by its theoretical value.

### DNA fragmentation assay

Excised mice liver was rinsed with ice-cold PBS twice and lysed in a buffer containing 10 mM Tris (pH 7.4), NaCl (150 mM), EDTA (5 mM), and Triton-X-100 (0.5%) for 30 min in ice-cold condition. Lysates were vortexed and centrifuged at 10,000 × g for 15 min. The supernatant containing DNA was extracted with equal volume of neutral phenol:chloroform:isoamyl alcohol (25:24:1). The purified DNA was electrophoresed using a 1% agarose gel containing ethidium bromide (1 μg mL^−1^). After checking the separation of DNA, images of the gels were captured using the AlphaImager Gel Documentation unit (Alpha Innotech, USA).

In addition to DNA fragmentation assay, histological investigation of liver excised from mice treated with 400 µg kg body wt^−1^ of MCYST-RR for 6 h was performed using Wright-Giemsa staining following standard procedure so as to assess the induction of apoptosis^52^. Apoptotic cells were identified on the basis of morphological features as described earlier.

### Pathway analysis

Graphs were drawn in Sigma Plot version 13 and GraphPad Prism version 5.04 for windows (www.graphpad.com). Heat Map analysis was done by Multi Experiment Viewer (MeV 4.9) software. Pathway analysis was done by; a) Reactome pathway database (http://www.reactome.org/), b) PANTHER (Protein ANalysis THrough Evolutionary Relationships) Classification System (http://www.pantherdb.org/), and c) DAVID Bioinformatics Resources version 6.7 (https://david.ncifcrf.gov/).

### Statistical analysis

All experiments were performed in triplicate under identical conditions. Mean values and standard deviations were determined from three replicates of each treatment. A one-way *ANOVA* (analysis of variance) ensured the significance of data according to Duncan’s multiple range test (*DMRT*) at *P* ≤ 0.05. Unless otherwise stated, values show the mean ± SD (*n* = 3).

## Electronic supplementary material

Supplementary information
