# Supplementary material for: Proteomic evidences for microcystin-RR-induced toxicological alterations in mice liver
Source: Sci Rep. 2018 Jan 22;8:1310. doi: 10.1038/s41598-018-19299-w (PMC5778043; doi:10.1038/s41598-018-19299-w)
Supplement: Supplementary file 1 — Supplementary information [file 41598_2018_19299_MOESM1_ESM.pdf]

Supplementary Information

Title of Article:

Proteomic Evidences for Microcystin-RR Induced Toxicological Alterations in Mice Liver

Authors: Ashutosh Kumar Rai, Rupesh Chaturvedi and Ashok Kumar

Supplementary Fig. S1- Peaks of eighteen protein spots along with its one typical peptide sequence. A-R showing peaks for spots 1 to 18 respectively. Amino acid sequence of a typical peptide is given along with its electropherogram.

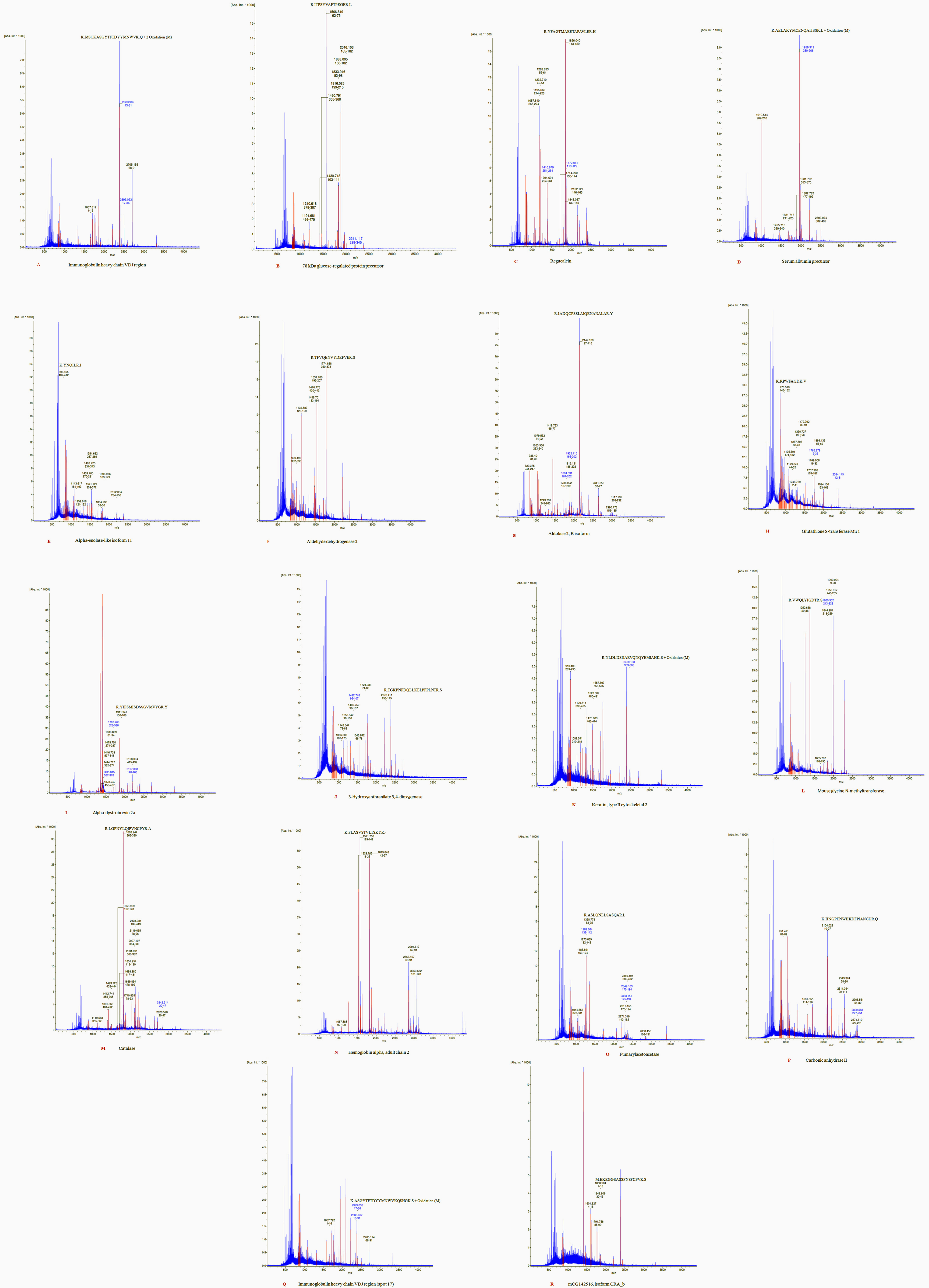
A Immunoglobulin heavy chain VDJ region
K.MSCKASGYTFTDYYMNWVK.Q + 2 Oxidation (M)
B 78 kDa glucose-regulated protein precursor
R.ITPSYVAFTPEGER.L
C Regucalcin
R.YFAGTMAEETAPAVLER.H
D Serum albumin precursor
R.AELAKYMCENQATISSK.L + Oxidation (M)
E Alpha-enolase-like isoform 11
K.YNQILR.I
F Aldehyde dehydrogenase 2
R.TFVQENVYDEFVER.S
G Aldolase 2, B isoform
R.IADQCPSSLAIQENANALAR.Y
H Glutathione S-transferase Mu 1
K.RPWFAGDK.V
I Alpha-dystrobrevin 2a
R.YIFSMISDSSGVMVYGR.Y
J 3-Hydroxyanthranilate 3,4-dioxygenase
R.TGKPNPDQLLKELPFPLNTR.S
K Keratin, type II cytoskeletal 2
R.NLDLDSIIAEVQNQYEMIAHK.S + Oxidation (M)
L Mouse glycine N-methyltransferase
R.VWQLYIGDTR.S
M Catalase
R.LGPNYLQIPVNCPYR.A
N Hemoglobin alpha, adult chain 2
K.FLASVSTVLTSKYR.-
O Fumarylacetoacetase
R.ASLQNLLSASQAR.L
P Carbonic anhydrase II
K.HNGPENWHKDFPIANGDR.Q
Q Immunoglobulin heavy chain VDJ region (spot 17)
K.ASGYTFTDYYMNWVKQSHGK.S + Oxidation (M)
R mCG142516, isoform CRA_b
M.EKEGGSASSFNSFCPVR.S

Supplementary Fig. S 2 (i & ii)**: Pathway Analysis**. Reactome (S 2-i), and PANTHER (S 2-ii) database analysis supports the occurrence of apoptosis as well as DNA fragmentation upon MCYST-RR treatment.

⇩ (Next page)

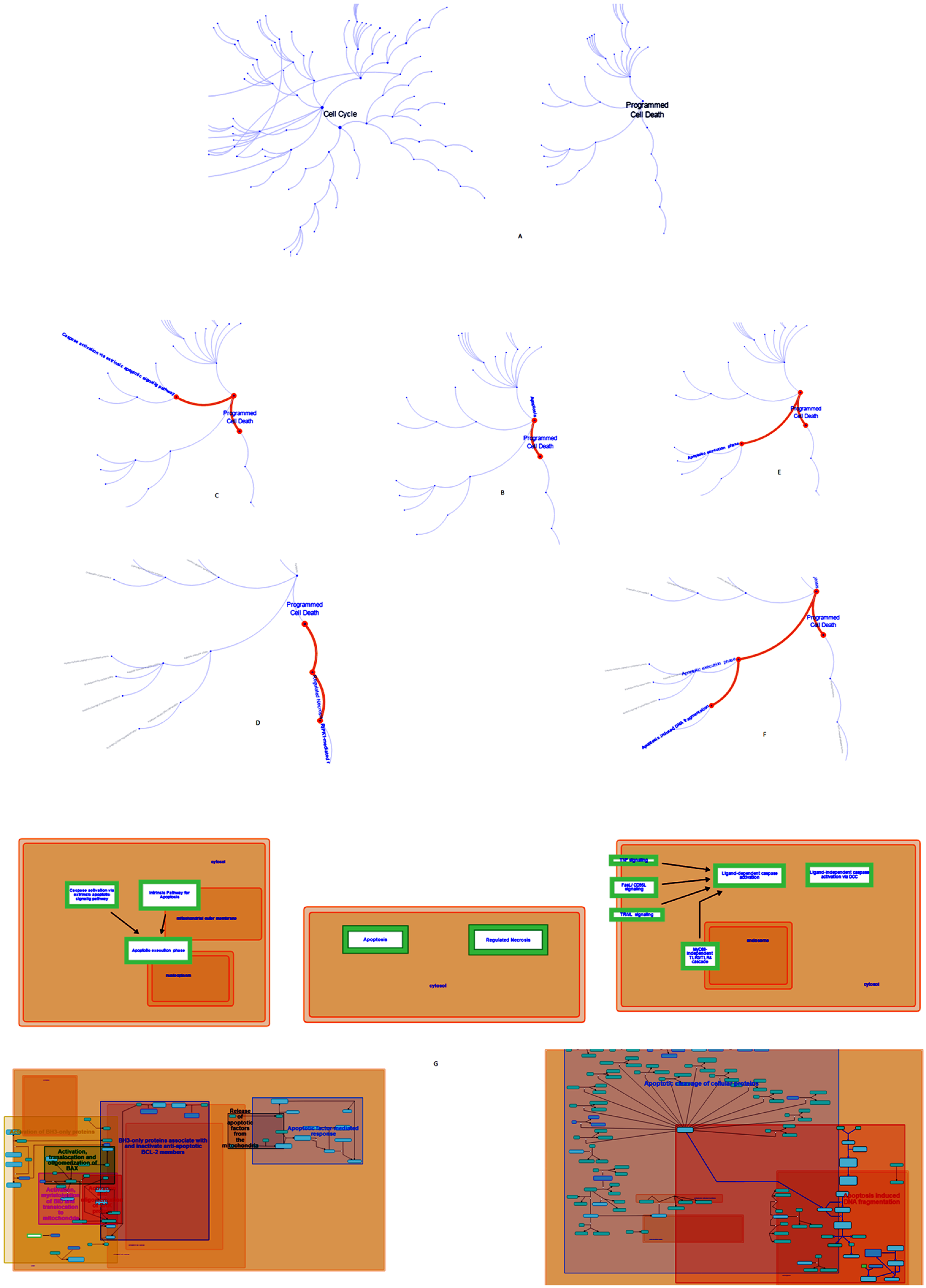

Supplementary Figure S 2 (i). Reactome Analysis

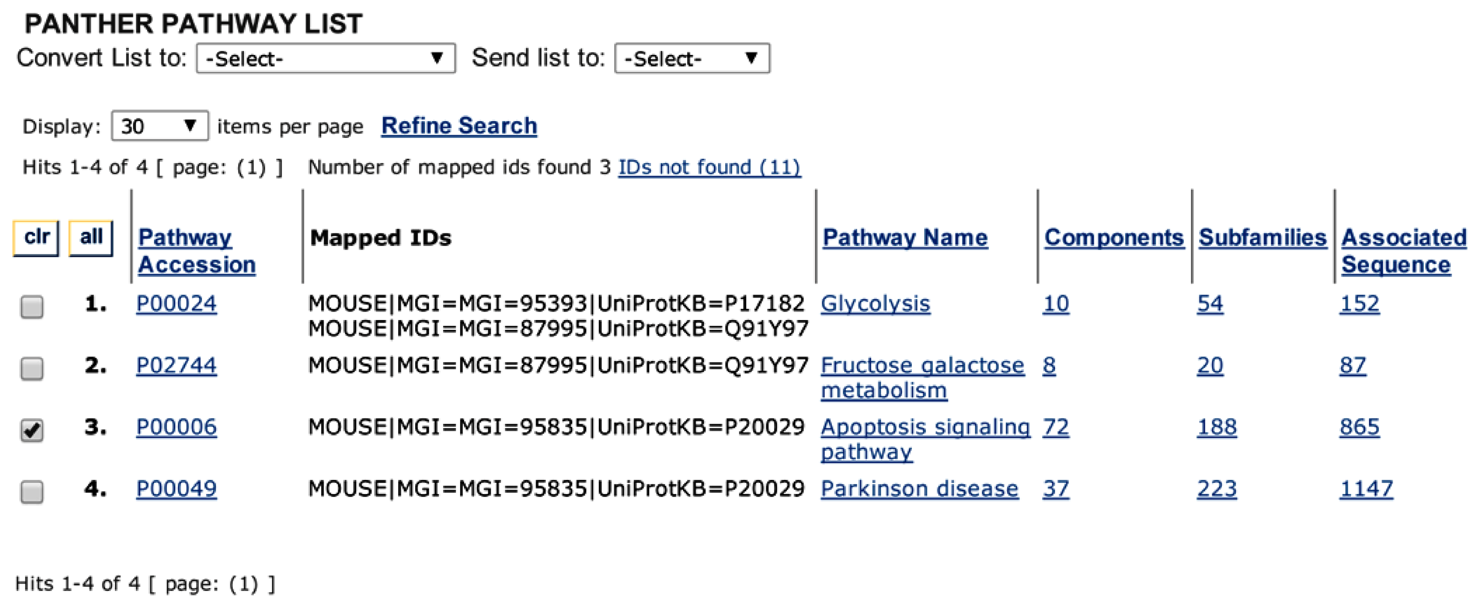

PANTHER PATHWAY LIST

Convert List to: -Select- Send list to: -Select-

Display: 30 items per page Refine Search

Hits 1-4 of 4 [page: (1)] Number of mapped ids found 3 IDs not found (11)

clr all		Pathway Accession	Mapped IDs	Pathway Name	Components	Subfamilies	Associated Sequence
☐	1.	P00024	MOUSE|MGI=MGI=95393|UniProtKB=P17182 MOUSE|MGI=MGI=87995|UniProtKB=Q91Y97	Glycolysis	10	54	152
☐	2.	P02744	MOUSE|MGI=MGI=87995|UniProtKB=Q91Y97	Fructose galactose metabolism	8	20	87
☑	3.	P00006	MOUSE|MGI=MGI=95835|UniProtKB=P20029	Apoptosis signaling pathway	72	188	865
☐	4.	P00049	MOUSE|MGI=MGI=95835|UniProtKB=P20029	Parkinson disease	37	223	1147

Hits 1-4 of 4 [page: (1)]

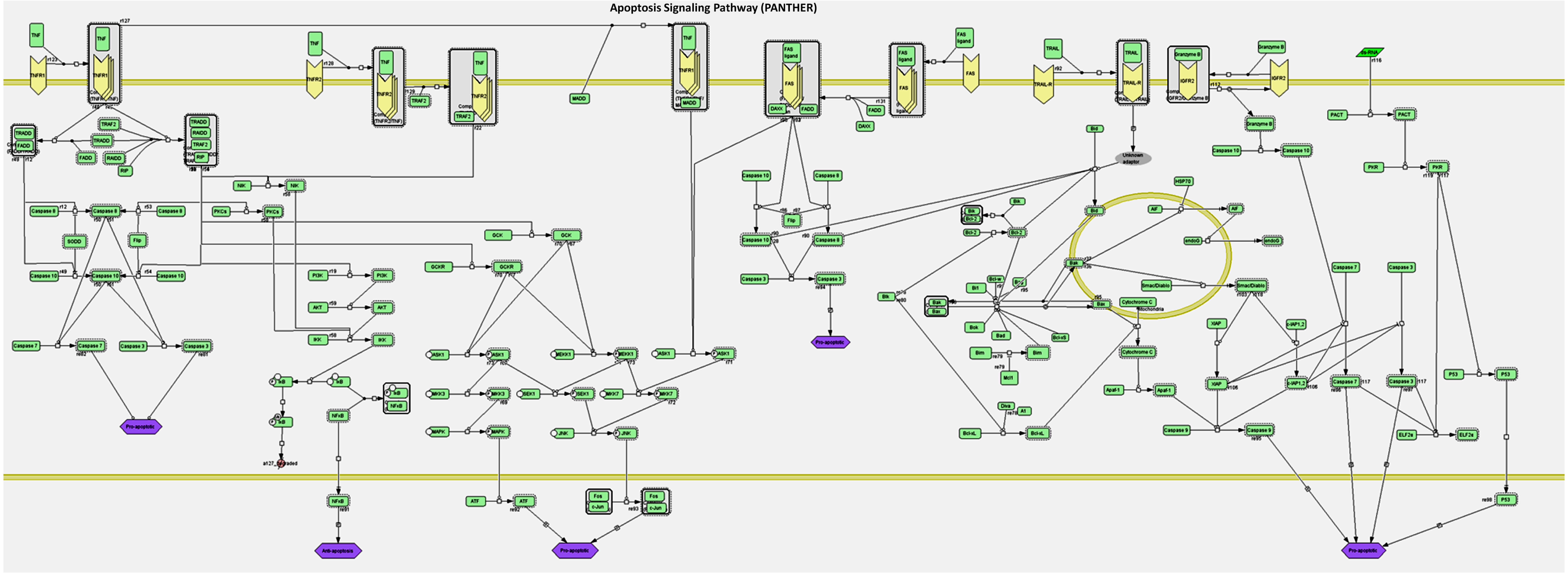

Supplementary Figure S 2 (ii).
